# Resurgence of *Bordetella pertussis* in Lazio: A Cross-Age Surveillance Study from Two Referral Hospitals

**DOI:** 10.3390/microorganisms13081808

**Published:** 2025-08-02

**Authors:** Giuseppe Sberna, Giulia Linardos, Eleonora Lalle, Rossana Scutari, Antonella Vulcano, Cosmina Mija, Licia Bordi, Barbara Bartolini, Fabrizio Maggi, Carlo Federico Perno, Carla Fontana

**Affiliations:** 1 Laboratory of Virology and Biosafety Laboratories, National Institute for Infectious Diseases Lazzaro Spallanzani—IRCCS, 00149 Rome, Italy; 2 Microbiology and Diagnostic Immunology Unit, Bambino Gesù Children’s Hospital, IRCCS, 00165 Rome, Italy; 3 Multimodal Laboratory Research Unit, Bambino Gesù Children’s Hospital, IRCCS, 00165 Rome, Italy; 4 Laboratory of Microbiology and Biological Bank, National Institute for Infectious Diseases Lazzaro Spallanzani—IRCCS, 00149 Rome, Italybarbara.bartolini@inmi.it (B.B.);

**Keywords:** *Bordetella pertussis*, adults, pediatric, co-infections

## Abstract

Since late 2023, an increase in *Bordetella pertussis* infections has been noticed in Europe, particularly among children. Our data showed the upward trend of *B. pertussis* cases in the Lazio region, even among adults with severe influenza-like illnesses, highlighting the necessity for maintaining high vaccination rates across both children and adults. These findings underscore the urgent need for clinicians to maintain a high index of suspicion for *B. pertussis* in patients with respiratory symptoms, prioritize nasopharyngeal swabs for accurate diagnosis, assess for co-infections, verify booster vaccination status in adults, and support timely reporting to public health authorities.

## 1. Introduction

*Bordetella pertussis* is the etiologic agent of pertussis (whooping cough), a highly contagious respiratory infection characterized by a cyclic epidemiological pattern, with peaks occurring every 3–5 years [1,2] due to immunity, whether naturally acquired or vaccine-induced, progressively declining over time, resulting in the accumulation of a new cohort of susceptible individuals [1,3]. *B. pertussis* has a worldwide distribution and affects individuals of all ages, being most severe in infants and young children [4,5]. In 2024, according to World Health Organization (WHO) and United Nations International Children’s Emergency Fund (UNICEF), 85% of children worldwide received the three doses of vaccine required for full immunization coverage [6]; in Europe, according to the latest European Centre for Disease Prevention and Control (ECDC) report, vaccination coverage among children varies across European countries, ranging from 84% to 99% [7], while Italy has a vaccination coverage exceeding 94% [8]. Despite the high vaccination coverage among children, since late 2023, an alarming increase in *B. pertussis* cases on the European continent has been observed, sparking significant public health concerns due to its potential to rapidly spread and cause serious complications, especially in vulnerable populations [7,9,10], like pneumonia, seizures, encephalopathy (brain inflammation or damage), and apnea (especially in infants). This increase may be attributed to multiple factors, such as reduced immunity following the COVID-19 pandemic, lower natural exposure to pathogens, as well as declining booster uptake, which leads to waning protection [7]; additionally, maternal vaccination, which plays a critical role in protecting newborns, is often suboptimal [7,10]. The increased number of cases of this disease has led to health authorities and researchers closely monitoring the situation in order to prevent further spread and to implement effective control measures [7]. In Italy, an outbreak affecting infants and young children emerged within the first five months of 2024, likely due to incomplete maternal immunization during pregnancy and suboptimal cocooning strategies, prompting crucial public health concerns [11,12]. This resurgence highlights the need for increased surveillance and timely intervention, as pertussis remains a significant cause of morbidity, particularly in unvaccinated or partially vaccinated individuals, who remain in these conditions as an outcome of a reluctance to vaccinate, missed scheduled immunizations, or insufficient awareness regarding booster dose recommendations for adults and adolescents. A comprehensive understanding of the epidemiology of the infection is crucial to optimizing public health interventions.

Based on these observations, in this dual-center study we examined whether there was also a rise in the identification of *B. pertussis* in respiratory specimens from adults and children experiencing severe influenza-like symptoms during 2024. We compared the incidence and epidemiological patterns in 2024 with data from previous years for both the pediatric and adult populations.

## 2. Materials and Methods

### 2.1. Adult Population

We retrospectively examined samples from patients with severe influenza-like symptoms, hospitalized in the Lazio region (Italy), and whose samples were sent to both the Laboratory of Virology and Microbiology at Rome’s National Institute for Infectious Diseases “Lazzaro Spallanzani” for urgent differential diagnosis. Patients had a median age of 58 years (range 18–99) and were predominantly male (62.3%). During the years 2019, 2020, 2021, 2022, 2023, and 2024, a total of 8669 samples from the respiratory tract were analyzed, among which 87.5% were nasopharyngeal swabs (NPS) and 12.5% were samples from the lower respiratory tract (such as bronchoalveolar lavages and sputum), as illustrated in Table 1. Data for the biological samples collected for diagnostic purposes were analyzed only after their anonymization.

The identification of respiratory pathogens was performed using three multiplex PCR methods, including the QIAstat-Dx Respiratory SARS-CoV-2 Panel (Qiagen s.r.l, Germantown, MD, USA [13]) and the BIOFIRE^®^ Respiratory Panel 2.1 plus (bioMérieux Clinical Diagnostics, Salt Lake City, UT, USA [14]), both of which directly amplify pathogens without the need for genomic extraction, while the third method required the extraction of samples with the QIAmp^®^ DNA Mini Kit on the QIAcube Connect (Qiagen [15]), followed by amplification with Allplex™ Respiratory Panel 4 (Seegene Inc., Seoul, Republic of Korea [16]) on the CFX96^TM^ Real-Time PCR System (Bio-Rad Laboratories S.r.l., Segrate, MI, Italia [17]).

Although three diagnostic platforms were employed, all methods were validated for *B. pertussis* detection and used consistently across the study period, ensuring comparability of results.

### 2.2. Pediatric Population

At the same time, patients aged 0–18 years with respiratory symptoms who were referred to Bambino Gesù Children’s Hospital in Rome, Italy, were examined for the presence of *B. pertussis*. A total of 3229 respiratory samples were collected and analyzed between January 2019 and December 2024. Most of these samples were nasopharyngeal aspirates (*n* = 2207, 80.7%), while the remainder were NPS (*n* = 1022, 19.3%; Table 2).

*B. pertussis* identification was evaluated by means of a single target real-time PCR for *B. pertussis* (Bordetella R-gene^®^, bioMérieux, Marcy-l’Étoile, France) according to the manufacturer’s recommendations [18].

Several other pathogens were identified by molecular and culture assays. An Allplex Respiratory Panel 4 (Seegene, Seoul, Republic of Korea [16]) and a BioFire FilmArray Respiratory Panel (bioMérieux Clinical Diagnostics, Salt Lake City, UT, USA [14]) were primarily used to identify the presence of respiratory viruses.

### 2.3. Statistical Analyses

The chi-square test and Fisher’s exact test were used to compare proportions of *B. pertussis* positivity across years and between sample types.

In addition to chi-square/Fisher’s exact tests, we performed further inferential analyses to assess temporal trends and potential predictors of *B. pertussis* positivity. A Cochran–Armitage trend test was applied separately to the adult and pediatric datasets to evaluate whether the observed increase in positivity rates was statistically significant over the years. For this purpose, the year variable was treated as ordinal.

Furthermore, a logistic regression model was constructed using individual-level data derived from aggregated counts. The binary outcome variable was *B. pertussis* positivity (positive vs. negative), and the predictors included year (as a continuous variable) and age group (adult vs. pediatric). This model aimed to estimate the odds of positivity over time and between age groups, adjusting for potential confounding.

Statistical analyses were performed using Python (version 3.13.5), including basic tests and regression modeling.

## 3. Results

**Figure 1:**
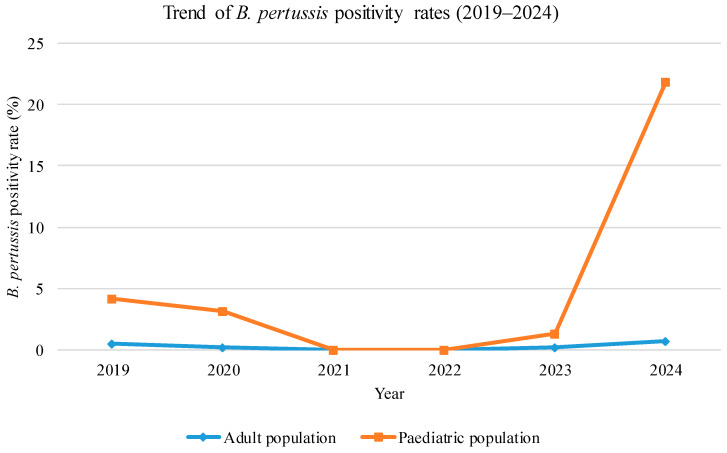
Percentage of positive samples for *Bordetella pertussis* coming from adult and pediatric populations in 2024.

**Figure 2:**
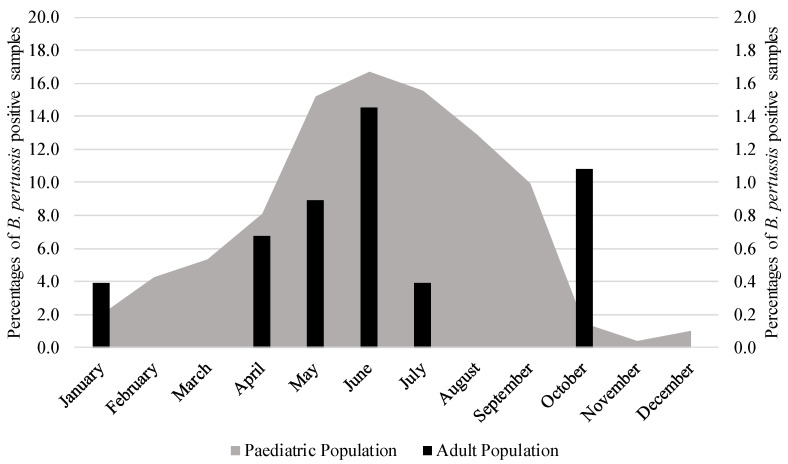
Percentage of *Bordetella pertussis*-positive samples from adult and pediatric populations in 2024.

Although numerous samples were examined during the evaluation period, *B. pertussis* detection was infrequent in the adult population in 2019 (3/555 cases, 0.5%), 2020 (4/2018 cases, 0.2%), and 2023 (3/1320 cases, 0.2%), with no cases observed in 2021 and 2022. There was an increase in the number of positive samples in 2024 (12/1763 cases, 0.7%), as shown in Table 1 and Figure 1 and Figure 2.

There was also a low rate of positivity for *B. pertussis* in children between 2019 and 2023 compared to the numbers seen in 2024. Specifically, 44/1051 cases (4.2%) were observed in 2019, 17/534 cases (3.2%) in 2020, no cases in 2021 (out of 267 tests) and 2022 (out of 210 tests), and 3/3221 cases (1.4%) in 2023. However, in 2024, a notable increase in positivity was observed, with 21.9% (207 out of 946 cases) testing positive, particularly during peaks in May and September (30.8%, 172/559 cases), as shown in Figure 1 and Figure 2 and in Table 2. The chi-square test or Fisher’s exact test (as appropriate) confirmed that the increase in 2024, compared to the other years examined, is statistically significant for both NPS (*p* < 0.05) and LRT (*p* < 0.05). Most of the positive results observed during this period were from patients younger than five years old (median age [interquartile range]: 4.44 [1.08–7.78] years).

Moreover, there was an uptick in the proportion of viral and/or bacterial co-infections found in those positive for *B. pertussis* in 2024 (37.5%) compared to the preceding four years (25.0%, 0.0%, 0.0%, and 33.3%), which could indicate increased complexity in treating these patients.

Regarding the distribution of co-infections in the pediatric population over the study period, we observed rates of 46.6% in 2019 and 52.9% in 2020, compared to 0% in 2023. Considering that data is available for 59/207 patients in 2024, the co-infection rate is 29.4%, with rhinovirus/enterovirus (33.3%) and *Mycoplasma pneumoniae* (33.3%) being the most commonly involved pathogens. Compared to the adult population examined, the rate of co-infections found in the pediatric population in 2024 is lower. This could be due to the lack of comprehensive co-infection information for pediatric patients positive for *B. pertussis*.

Our retrospective study highlights that in the adult population, *B. pertussis* was mainly detected in NPS, since only one positivity was revealed in different biological fluids (Table 1). Concerning the pediatric population, in 2019 and 2020, the majority of *B. pertussis*-positive samples originated from the lower respiratory tract (81.8% and 88.2%, respectively), while a marked shift was observed in 2024, with the majority of positive samples being NPS (72.0%; Table 2). Furthermore, a significant difference was found between the number of positive NPS samples compared to LRTS (*p* < 0.05).

### Trend and Regression Analysis

The Cochran–Armitage trend test revealed a statistically significant upward trend in *B. pertussis* positivity rates over time in both adults (Z = 2.14, *p* = 0.032) and pediatric patients (Z = 5.87, *p* < 0.001), confirming the descriptive observation of increased incidence in 2024.

Logistic regression analysis further supported these findings. The model showed that each additional year was associated with a significant increase in the odds of testing positive for *B. pertussis* [Odds ratio (OR) = 1.32, 95% Confidence Interval (CI): 1.18–1.47, *p* < 0.001]. Additionally, pediatric patients had significantly higher odds of positivity compared to adults (OR = 4.85, 95% CI: 3.92–5.99, *p* < 0.001) after adjusting for year.

## 4. Discussion

Since late 2023, Europe has experienced a significant increase in *B. pertussis* infections, raising concerns about potential public health impacts worldwide. This increase has been particularly evident among children in the first few months of 2024 [11,12]. Several EU regions have reported notable resurgences [11]. Spain has documented a marked uptick in cases, especially among infants and adolescents with incomplete vaccination coverage [19]. In Denmark, public health authorities have noted clusters of outbreaks linked to waning immunity and delays in booster vaccinations [20]. In England, provisional data from the UK Health Security Agency show that 14,894 pertussis cases were confirmed by laboratory testing in 2024. The monthly figures climbed sharply, from 554 in January to over 3000 by May 2024 [2]. An increase in *B. pertussis* incidence has also been reported in France, with more than 5000 laboratory-confirmed cases identified between January and May 2024 [21].

Our findings suggest an increase in *B. pertussis* infections in the Lazio region, including among adults exhibiting serious influenza-like symptoms. Statistical analyses reinforce the robustness of our findings. The significant trend detected by the Cochran–Armitage test confirms that the increase in *B. pertussis* positivity observed in 2024 is not due to random variation but reflects a true epidemiological shift. The logistic regression model highlights the disproportionate burden of infection among pediatric patients and the progressive rise in positivity over time. This indicates a rise compared to previous years. The greater occurrence of co-infections could lead to more severe illness and potentially more emergency room visits. A previous study confirmed a high rate of *B. pertussis* co-infection, particularly among younger patients. There was a higher prevalence among patients with respiratory failure respect to those without: 92.9% versus 69.0% [12]. As previously described by Gan et al., it is important to note that co-infections may be linked to more severe respiratory symptoms and greater utilization of healthcare [22]. In our study, the presence of co-infections was more frequent in adults than in children, potentially indicating a more complex clinical course.

It is also noteworthy that most of the samples that tested positive for *B. pertussis* in this study were collected using NPS, which supports the current guidelines of the Italian National Institute of Health [23,24]. These guidelines recommend NPS as the optimal diagnostic tool for this bacterium; moreover, discrepancies in positivity rates across different sample types are likely attributable to variations in diagnostic sensitivity. NPS are considered the gold standard for detecting *B. pertussis*, especially in the early stages of infection. In contrast, lower detection rates may be observed in samples from the lower respiratory tract, potentially due to a diminished bacterial load or inconsistencies in sampling techniques.

Furthermore, the population’s growing vulnerability to infections from *B. pertussis* may be partially attributed to the relaxation of COVID-19 containment measures, although further studies are needed to confirm this association. Our research highlighted an exceptionally low incidence of *B. pertussis* during 2020 and 2023, and no recorded cases in 2021 and 2022, with a notable increase in early 2024. In line with current clinical recommendations [25], the prompt initiation of macrolide antibiotics (i.e., azithromycin or clarithromycin) is advised to limit disease transmission and alleviate symptom severity; post-exposure prophylaxis is recommended for close contacts, particularly in households with vulnerable individuals.

Concerning the increased percentage of cases among both the adult and the pediatric population of the Lazio region revealed in this study, it is crucial to raise public awareness about the importance of vaccination, despite the high pediatric vaccination coverage reported in Italy (94.8%) [8], as well as in the Lazio region (94.9%) [8]. Comprehensive vaccination coverage needs to be ensured, not only for children but also extending vaccination efforts to the adult population, reminding the adult population the importance of undergoing the recommended periodic booster vaccinations. Indeed, according to the Italian Ministry of Health, adult boosters should be administered every 10 years following the previous dose [26]. These measures could be fundamental in controlling the spread of *B. pertussis* and safeguarding overall community health.

Additionally, public health strategies should include continuous surveillance and the timely reporting of *B. pertussis* cases to detect and respond to outbreaks promptly. Health educational campaigns emphasizing the importance of booster vaccinations for adults, especially those in close contact with infants and young children, could further mitigate the spread of the infection. Collaboration between healthcare providers, public health authorities, and the community is essential to enhance vaccination rates and protect vulnerable populations.

Collaboration between the National Institute for Infectious Diseases “Lazzaro Spallanzani” and Bambino Gesù Children’s Hospital enabled comprehensive surveillance spanning all ages, combining complementary diagnostic expertise and coordinated data collection. This synergy enabled the early detection of demographic shifts in pertussis incidence and the emergence of complex co-infection patterns, thereby reinforcing the importance of integrated public health strategies. The findings emphasize the urgent need to strengthen vaccination coverage across all age groups, as well as the importance of implementing continuous, collaborative surveillance systems as a model for effective outbreak response and prevention.

## 5. Limitations of This Study

Despite the fact that this study offers important insights into the recent resurgence of *B. pertussis* in Lazio, some limitations should be noted. Individual-level vaccination data were not available, which limited our ability to explore associations between immunization status and infection risk. The retrospective design and lack of stored isolates also prevented the molecular characterization of circulating strains. Additionally, clinical data were incomplete for some patients, particularly those referred from external facilities. Finally, as the study was conducted in two referral hospitals, findings may not fully reflect the broader regional or national context. Nonetheless, these limitations are common in retrospective surveillance studies and highlight the need for future prospective research with more granular data.

## Figures and Tables

**Table 1:** Multiplex PCR results of nasopharyngeal swabs (NPS) and lower respiratory tract samples (LRTS, i.e., bronchoalveolar lavages and sputum) coming from hospitalized adult patients with severe influenza-like symptoms from 2019 to 2024.

	2019		2020		2021		2022		2023		2024	
	NPS ^1^	LRTS ^2^	NPS	LRTS	NPS	LRTS	NPS	LRTS	NPS	LRTS	NPS	LRTS
	P ^3^/T ^4^	P/T	P/T	P/T	P/T	P/T	P/T	P/T	P/T	P/T	P/T	P/T
January	1/80	0/16	1/158	0/10	0/92	0/10	0/76	0/14	0/159	0/25	1/168	0/53
February	0/74	0/22	0/332	0/7	0/170	0/11	0/62	0/17	2/151	0/17	0/34	0/39
March	0/55	0/8	2/398	0/11	0/307	0/10	0/83	0/17	1/102	0/19	0/60	0/46
April	0/30	0/10	0/224	0/6	0/139	0/12	0/90	0/17	0/83	0/18	1/65	0/45
May	0/23	0/12	1/213	0/14	0/109	0/9	0/52	0/35	0/81	0/20	2/99	0/38
June	1/25	0/7	0/78	0/1	0/87	0/15	0/38	0/13	0/70	0/15	4/113	0/19
July	0/13	0/3	0/85	0/9	0/63	0/13	0/63	0/9	0/68	0/7	1/106	0/9
August	0/10	0/4	0/29	0/4	0/62	0/7	0/56	0/14	0/64	0/17	0/112	0/22
September	0/18	0/1	0/97	0/3	0/52	0/12	0/55	0/13	0/60	0/11	0/116	0/20
October	0/32	0/6	0/114	0/1	0/71	0/12	0/91	0/7	0/66	0/5	2/154	1/18
November	0/44	0/1	0/101	0/0	0/106	0/9	0/76	0/12	0/86	0/16	0/145	0/24
December	1/54	0/7	0/123	0/0	0/93	0/9	0/106	0/15	0/143	0/17	0/237	0/21
Total	3/458	0/97	4/1952	0/66	0/1351	0/129	0/848	0/183	3/1133	0/187	11/1408	1/355
	3/555		4/2018		0/1480		0/1031		3/1320		12/1763	

^1^ NPS: nasopharyngeal swabs; ^2^ LRTS: lower respiratory tract samples; ^3^ P: number of positive results; ^4^ T: total number of samples tested.

**Table 2:** Multiplex PCR results of nasopharyngeal swabs (NPS) and lower respiratory tract samples (LRTS, i.e., bronchoalveolar lavages and sputum) collected from pediatric patients with severe influenza-like symptoms from 2019 to 2024.

	2019		2020		2021		2022		2023		2024	
	NPS ^1^	LRTS ^2^	NPS	LRTS	NPS	LRTS	NPS	LRTS	NPS	LRTS	NPS	LRTS
	P ^3^/T ^4^	P/T	P/T	P/T	P/T	P/T	P/T	P/T	P/T	P/T	P/T	P/T
January	0/5	4/208	0/28	8/133	0/1	0/7	0/0	0/18	0/6	0/37	0/7	5/29
February	0/4	1/152	2/57	3/83	0/1	0/12	0/0	0/12	0/1	0/11	0/1	4/20
March	1/6	3/121	0/30	2/42	0/3	0/10	0/6	0/9	0/3	0/18	1/8	7/36
April	0/1	6/81	0/10	1/13	0/1	0/8	0/0	0/12	0/1	0/12	3/13	9/25
May	0/2	5/62	0/0	0/5	0/0	0/17	0/1	0/12	0/3	0/13	23/58	11/29
June	0/1	3/43	0/4	1/17	0/0	0/11	0/2	0/6	0/1	0/8	38/115	8/29
July	1/3	4/20	0/2	0/11	0/2	0/6	0/1	0/3	0/7	0/6	38/122	2/20
August	4/6	2/21	0/1	0/21	0/1	0/7	0/0	0/6	1/6	1/5	22/66	7/25
September	1/3	3/30	0/5	0/15	0/1	0/4	0/1	0/9	0/8	0/10	17/73	6/22
October	0/2	3/36	0/3	0/18	0/9	0/22	0/1	0/14	1/10	0/5	4/87	0/18
November	0/13	0/63	0/1	0/18	0/25	0/64	0/5	0/25	0/3	0/11	1/71	0/22
December	1/28	2/140	0/2	0/15	0/19	0/35	0/17	0/50	0/10	0/26	2/26	1/24
Total	8/74	36/977	2/143	15/391	0/64	0/203	0/34	0/176	2/59	1/162	149/648	58/298
	44/1051		17/534		0/267		0/210		3/221		207/946	

^1^ NPS: nasopharyngeal swabs; ^2^ LRTS: lower respiratory tract samples; ^3^ P: number of positive results; ^4^ T: total number of samples tested.

## Data Availability

The original contributions presented in this study are included in the article. Further inquiries can be directed to the corresponding author.
